# A germline mutation in the *BRCA1* 3’UTR predicts Stage IV breast cancer

**DOI:** 10.1186/1471-2407-14-421

**Published:** 2014-06-10

**Authors:** Jemima J Dorairaj, David W Salzman, Deirdre Wall, Tiffany Rounds, Carina Preskill, Catherine AW Sullivan, Robert Lindner, Catherine Curran, Kim Lezon-Geyda, Terri McVeigh, Lyndsay Harris, John Newell, Michael J Kerin, Marie Wood, Nicola Miller, Joanne B Weidhaas

**Affiliations:** 1 Discipline of Surgery, School of Medicine, National University of Ireland, Galway, Ireland; 2 Department of Therapeutic Radiology, Yale School of Medicine, New Haven, CT 06510, USA; 3 HRB Clinical Research Facility, National University of Ireland, Galway, Ireland; 4 School of Mathematics, Statistics and Applied Mathematics, National University of Ireland, Galway, Ireland; 5 Department of Medicine, University of Vermont, Burlington, VT 05405, USA; 6 Department of Medicine, Yale School of Medicine, New Haven, CT 06510, USA; 7 Institute of Pharmacy and Molecular Biotechnology, University of Heidelberg, Heidelberg, Germany

**Keywords:** *BRCA1-*3’UTR-variant, Mutation, Breast cancer, Stage IV breast cancer, Metastatic breast cancer, Biomarker, Diagnostic marker

## Abstract

**Background:**

A germline, variant in the *BRCA1* 3’UTR (rs8176318) was previously shown to predict breast and ovarian cancer risk in women from high-risk families, as well as increased risk of triple negative breast cancer. Here, we tested the hypothesis that this variant predicts tumor biology, like other 3’UTR mutations in cancer.

**Methods:**

The impact of the *BRCA1-*3’UTR-variant on *BRCA1* gene expression, and altered response to external stimuli was tested *in vitro* using a luciferase reporter assay. Gene expression was further tested *in vivo* by immunoflourescence staining on breast tumor tissue, comparing triple negative patient samples with the variant (TG or TT) or non-variant (GG) *BRCA1* 3’UTR. To determine the significance of the variant on clinically relevant endpoints, a comprehensive collection of West-Irish breast cancer patients were tested for the variant. Finally, an association of the variant with breast screening clinical phenotypes was evaluated using a cohort of women from the High Risk Breast Program at the University of Vermont.

**Results:**

Luciferase reporters with the *BRCA1-*3’UTR-variant (T allele) displayed significantly lower gene expression, as well as altered response to external hormonal stimuli, compared to the non-variant 3’UTR (G allele) in breast cancer cell lines. This was confirmed clinically by the finding of reduced *BRCA1* gene expression in triple negative samples from patients carrying the homozygous TT variant, compared to non-variant patients. The *BRCA1-*3’UTR-variant (TG or TT) also associated with a modest increased risk for developing breast cancer in the West-Irish cohort (OR = 1.4, 95% CI 1.1-1.8, *p* = 0.033). More importantly, patients with the *BRCA1-*3’UTR-variant had a 4-fold increased risk of presenting with Stage IV disease (*p* = 0.018, OR = 3.37, 95% CI 1.3-11.0). Supporting that this finding is due to tumor biology, and not difficulty screening, obese women with the *BRCA1-*3’UTR-variant had significantly less dense breasts (p = 0.0398) in the Vermont cohort.

**Conclusion:**

A variant in the 3’UTR of *BRCA1* is functional, leading to decreased *BRCA1* expression, modest increased breast cancer risk, and most importantly, presentation with stage IV breast cancer, likely due to aggressive tumor biology.

## Background

Breast cancer is the third most common form of cancer, with almost 1.5 million women in the world diagnosed with the disease in 2010 alone [1,2]. The extensive use of mammography has resulted in a large proportion of breast cancer cases being detected at an earlier stage, resulting in increased survival and outcome [3]. However, approximately 3-6% of patients continue to present with metastatic disease at diagnosis throughout the US and Europe [4,5]. As a significant number of cases present with metastatic disease when the primary tumor is not locally advanced [6], one can hypothesize that there is heterogeneity in tumor biology between patients, versus a failure of screening. Despite the presence of targeted therapeutics for hormone receptor sensitive and HER2 over-expressing breast cancers, treatment of metastatic disease remains incurable. Therefore, identification of women with a predisposition to develop tumors that are more likely to metastasize is critical to help develop improved prevention and screening strategies for those individuals.

The Breast Cancer 1, early onset gene (*BRCA1*) located on chromosome 17q21.31 [7,8] encodes a tumor suppressor that plays a critical role in the DNA damage response and repair pathways [9,10]. Germline variants in the open-reading-frame of *BRCA1* confer a mean risk of 54% and 39% for developing hereditary breast and ovarian cancer (respectively) by age 70 [11-14]. However, *BRCA1* open-reading-frame variants only account for a small portion of hereditary breast cancer cases that occur primarily in young, premenopausal patients [15]. Therefore, the search for additional germline variants, outside of the *BRCA1* open-reading-frame predicting increased breast cancer risk has been undertaken. Such variants in the *BRCA1* 3’UTR have recently been identified and were first implicated in breast and ovarian cancer susceptibility in high-risk families [16]. Two variants *5711 + 421 T/T* and *5711 + 1286 T/T* (located in the *BRCA1* 3’UTR) are associated with cancer risk in Thai women from breast and ovarian cancer families (OR = 3.0). Independent evaluation of the *5711 + 421 T/T* variant (referred to here as rs8176318 or the *BRCA1-*3’UTR-variant) revealed significant variation in baseline frequency by ethnicity, with a documented minor allele frequency in Irish populations of approximately 0.28 [17]. Homozygous G > T variants were found to be associated with increased risk of breast cancer in African American women (OR = 9.48, 95% CI 1.01-88.80), and were specifically associated with the development of triple negative breast cancer (OR = 12.19, 95% CI 1.29-115.21) [17]. This data suggests that the *BRCA1-*3’UTR-variant not only confers an increased risk of developing breast cancer, but may also be associated with tumor biology, since the propensity to develop triple negative breast cancer is higher than that of the other subtypes. One could hypothesize from these findings that the *BRCA1-*3’UTR-variant functions similarly to that of canonical *BRCA1* open-reading-frame variants, which are more commonly associated with development of triple negative breast cancer as opposed to the other subtypes [18-20].

Open reading frame variants in *BRCA1* have not clearly been associated with unique tumor biology, but only have been predictive of response to therapeutic agents that take advantage of their inherent DNA repair defects [21]. In contrast, 3’UTR variants in cancer- associated genes have been shown to predict both altered response to specific therapies, as well as inherent differences in tumor biology. This is likely due to the fact that these variants are in regions of regulatory elements that control the nature and timing of gene expression, and their effects are only manifest under particular extracellular and/or intracellular stimuli (for review see ([22]). One mechanism for regulation of these variants is by trans-acting factors such as miRNAs, which are rapidly altered by external factors such as genotoxic stress [23] and estrogen receptor signaling [24].

Based on evidence of the biological function of other 3’UTR variants in cancer, and the association of the *BRCA1-*3’UTR-variant with breast cancer risk in two previous studies [16,17], we sought to investigate the impact of this variant on *BRCA1* expression and its association with tumor biology as seen in clinical presentation in a clinically well-annotated breast cancer population.

## Methods

### Luciferase reporter assay

Luciferase reporters containing either the rs8176318 G-allele or T-allele were generated by PCR amplification of the *BRCA1* 3’UTR loci from HMEC genomic DNA (heterozygous for the *BRCA1-*3’UTR-variant) using the following DNA oligonucleotides:

*BRCA1* forward 5’ ATGACTCGAGCTGCAGCCAGCCACAGGTACAGAGCCACAG 3’

*BRCA1* reverse 5’ ATGAGCGGCCGCGTGTTTGCTACCAAGTTTATTTGCAGTG 3’

PCR amplicons were subcloned into the XhoI and NotI sites (underlined) of the psiCHECK2 dual luciferase vector (Progema). Constructs were sequence verified to confirm that the only difference in the *BRCA1* 3’UTR was the rs8176318 variant.

MCF-7, MDA-MB-231, MDA-MB-361, MDA-MB-468, Hs 578 T and BT-20 cells were purchased from the ATCC and grown at 37°C and 5% CO_2_ according to the manufacturer’s protocol. MCF-7 and BT-20 cells were cultured using MEM (GIBCO) supplemented with 10% fetal bovine serum (GIBCO) and 100 ug/ml penicillin, 100 U streptomycin. MDA-MB-231, MDA-MB-361 and MDA-MB-468 cells were cultured using Leibovitz’s L-15 (GIBCO) supplemented with 10% fetal bovine serum and 100 ug/ml penicillin, 100 U streptomycin. Hs5788T cells were cultured in DMEM (GIBCO) supplemented with 10% fetal bovine serum and 100 ug/ml penicillin, 100 U streptomycin.

Cells in log-growth phase were transfected with either the G-allele or T-allele expressing luciferase reporters (100 ng) using Lipfectamine 2000 (Invitrogen) according to the manufacturer’s protocol. Following a 16-hour incubation the cells were lysed and analyzed for dual luciferase activities by quantitative titration using the dual luciferase assay kit (Promega). Renilla luciferase was normalized to firefly luciferase. Graphed is the mean ± standard deviation (SD) of 3 independent experiments. Statistical significance was determined by student’s t-test (1-tailed, paired t-test). A p-value of less than 0.05 was considered statistically significant.

### Immunofluorescence staining of *BRCA1* in tumor tissue

*BRCA1* protein expression was analyzed from tumor tissue derived from the triple negative breast cancer cohort subset with corresponding *BRCA1-*3’UTR-variant genotype information, using an immunofluorescent platform, AQUA™, on tissue microarrays (TMAs) of tumor cores. *BRCA1* protein was assessed using monoclonal MS110 Ab-1 anti-*BRCA1* (Calbiochem) [25-27] and rabbit polyclonal anticytokeratin (DAKO), at dilutions of 1:100 and 1:200 respectively in 0.3% BSA/TBS buffer for 1 h at 37°C. AQUA has been described previously [28,29].

### Estrogen withdrawal assay

MCF-7 cells cultured in phenol-red free MEM (GIBCO) containing 5% fetal bovine serum and 100 ug/ml penicillin, 100 U streptomycin, were treated with either 100 nM Fuvestrant (Sigma I4409) or β-Estradiol (Sigma E8875). Following a 48-hour incubation, the cells were transfected with luciferase reporters (100 ng) harboring either the *BRCA1* G-allele or T-allele 3’UTR using Lipofectamine 2000. After a 16-hour incubation the cells were lysed and analyzed for dual luciferase activities by quantitative titration. Renilla luciferase was normalized to firefly luciferase. Graphed is the mean ± SD of 3 independent experiments, preformed in triplicate. Statistical significance was determined by student’s t-test (1-tailed, paired t-test). A *p*-value of less than 0.05 was considered statistically significant.

Total RNA was isolated from cell lysates by Trizol extraction as previously described [30]. cDNA was generated using iScript cDNA Synthesis Kit (Bio-Rad). Target mRNA was amplified by qPCR using iTaq SYBR Green Supermix with ROX (Bio-Rad) on a 7900HT Fast Real-Time PCR System (Applied Biosystems) using the following DNA oligonucleotide primers:

*Actin* forward 5’ AGAAAATCTGGCACCACACC 3’

*Actin* reverse 5’ AGAGGCGTACAGGGATAGCA 3’

*GREB1* forward 5’ GTGGTAGCCGAGTGGACAAT 3’

*GREB1* reverse 5’ TGTGCATTACGGACCAGGTA 3’

*TFF1* forward 5’ CACCATGGAGAACAAGGTGA 3’

*TFF1* reverse 5’ CCGAGCTCTGGGACTAATCA 3’

mRNA levels were calculated by the delta-delta C_T_ method [31]. Samples were run in triplicate and standard deviation (SD) is the average of 3 independent experiments.

### Study populations

All women with a biopsy confirming breast cancer at Galway Hospital and its affiliates are approached to enroll in the breast cancer study including DNA collection. Informed consent, a detailed family history of breast and/or ovarian cancer and a peripheral venous blood sample are obtained from cases and controls. Controls were women from the west of Ireland, primarily over 60 years of age, without a personal history of cancer of any type and without a first-degree family member with breast or ovarian cancer. These controls were accrued primarily from Active Retirement association meetings and from Nursing home residents. All cases and controls were recruited following appropriate ethical approval from the Galway University Ethics Committee. 728 cases and 387 controls were included from this cohort.

The Irish patient cohort consisted of 728 women with invasive, primary operable breast cancer diagnosed between June 1980 and August 2007, with complete receptor status (outlined in Additional file 1). Receptor status was determined using established histopathological methods and immunohistochemistry, followed by fluorescence in-situ hybridisation (FISH) to confirm HER2/*neu* positivity in samples that scored a 2+ on Hercept test. The samples were then grouped into Luminal A, Luminal B, HER2 and triple negative subtypes based on receptor status but in the absence of gene expression analysis. Patient demographics and tumor characteristics were recorded and outcome/survival data was prospectively maintained using hospital medical records. Disease free survival (DFS) was defined as time in months, from breast cancer diagnosis to point of loco/regional recurrence or distant disease progression, progression free survival (PFS) was defined as time in months from the point of diagnosis of Stage IV cancer to disease progression and overall survival (OS) was defined as the time from breast cancer diagnosis to the end of follow-up or death (months).

The CT Triple Negative Breast Cancer (TNBC) Cohort has been previously described [32], but briefly, FFPE tissue was obtained from 134 TNBC patients, who underwent surgery at the Yale University New Haven Hospital or the Hospital of Bridgeport, Connecticut, between 1985 and 2007. Patient sample collection was performed through a Yale HIC approved tissue collection protocol. Tissue of 120 patients was used for TMA construction and the follow up time for these patients ranged between 3 months and 19 years with a mean follow up of 4.4 years. Patient age at diagnosis ranged from 30 to 90 years, with a mean age at diagnosis of 53 years. Sixty-two patients were diagnosed as node negative and 40 patients as node positive. There were 65 patients who were Caucasian in this cohort who were used for this analysis. Treatment was known in 86% of patients, out of those 63% received chemotherapy. Gene expression in TMAs was analyzed by AQUA technology [28,29], and results were reviewed and confirmed by two independent pathologists.

The High Risk Breast Program from Vermont is a database that is IRB approved and was established at the University of Vermont in 2003. Eligible women include those with a strong family history of breast cancer (55.2% of participants), a prior breast biopsy showing atypical ductal hyperplasia or lobular neoplasia (14.7%), a known germline abnormality of *BRCA1* or 2 (7.3%, but excluded from this study), or a prior history of receiving chemo-radiotherapy for Hodgkin’s disease (1.3%). At study entry, unaffected high-risk women provide anthropometric measurements, medical/family history, physical activity and diet information, mammography reports, health behavior information and provide a blood sample for storage that may be used for future research. 536 women have been enrolled into this database since 2003 with follow-up visits, questionnaire completion and blood draws occurring at 4 and 8 years after study entry. Status of enrolled women (i.e., new cancer diagnosis) is updated on an ongoing annual basis. For this study, 367 women were genotyped for the *BRCA1-*variant.

### *BRCA1-*3’UTR-variant genotyping

1–3 mL of whole blood was drawn from the Irish cases and controls and DNA was isolated. DNA was isolated from FFPE tissue for genotyping for the TNBC Cohort. DNA was supplied from the Vermont cohort. From blood, DNA was isolated using a DNA extraction kit (Gentra Puregene) or Ambion according to the manufacturer’s protocol. Genotyping was performed using a custom TaqMan genotyping assay (Applied Biosysytems) that was specific for rs8176318. Each reaction was performed in a 20 μl volume using 10 μl of 2× TaqMan Genotyping MaterMix, 1 μl of the 20× variant assay, approximately 40 ng of DNA and nuclease free water in a 96-well plate. The reactions were run on the Applied Biosystems 7900HT Fast Real-Time PCR System in a two-stage process incorporating PCR amplification and allelic discrimination. Genotypes were analyzed using the Applied Biosystems SDS 2.3 genotyping software and automatic calls were verified by observing the spectral contributions of the dye corresponding to the sequence specific probe on the Multicomponent Data Plot. Internal quality control was maintained using established positive and negative controls to ensure genotyping accuracy and 6% percent of DNA samples were genotyped in duplicate with 100% consistency of results. Two DNA samples of the 728 cases failed to amplify and were excluded from further analyses. All Caucasian cases from the TNBC cohort amplified and were included in the analysis. All BRCA coding sequence non-mutant patients from the Vermont cohort were included.

### Statistical analysis

The genetic distribution of the breast cases and controls were tested for Hardy-Weinberg equilibrium and were found to be in equilibrium. In order to evaluate the distribution of patient demographics in cases and controls as well as tumor features among the cases, categorical variables were analyzed using the χ^2^ test and continuous variables were analyzed using t-tests. Binary logistic regression was used to evaluate the association of each genotype with cancer. Case–control analysis comparing genotypes in different models was performed using a χ^2^ test to obtain odds ratios (OR), 95% Confidence Interval (CI) and p-values. Based on the preceding statistical findings, the dominant model was used for all further analyses.

Prevalence of the variant across cancer subtypes, and comparison of the respective subtypes against controls were evaluated using χ^2^ analyses. The Luminal A cases were stratified according to menopausal status and the observed genotype distribution compared with controls using χ^2^ test. Association of the variant with ER/PR status controlling for other patient and tumor variables was analyzed using binary logistic regression.

Binary logistic regression was used to evaluate the independent effect of metastasis and disease stage in predicting variant positivity in all cancer cases and Luminal A cases specifically. Logistic regression analyses for all cases and Luminal A cases with a binary outcome variable coded as rs8176318 positive (TT or GT genotypes) or negative (GG genotype) included variables such as age at diagnosis, menopausal status, tumor grade, ER/PR status and stage.

Cox Proportional Hazards models were fitted to evaluate the effect of the variant on disease free survival, progression free survival and overall survival in all cancer cases and according cancer stage.

Fisher’s Exact Test was used to examine the statistical significance of the association between mammographic density and the presence or absence of the *BRCA1-*3’UTR-variant in both the entire population, as well as in a variety of subsets (BMI categories, pre- or post-menopausal women, and age at menarche categories).

## Results

### The BRCA1-3’UTR-variant is associated with decreased gene expression in triple negative breast cancer cell lines

**Figure 1:**
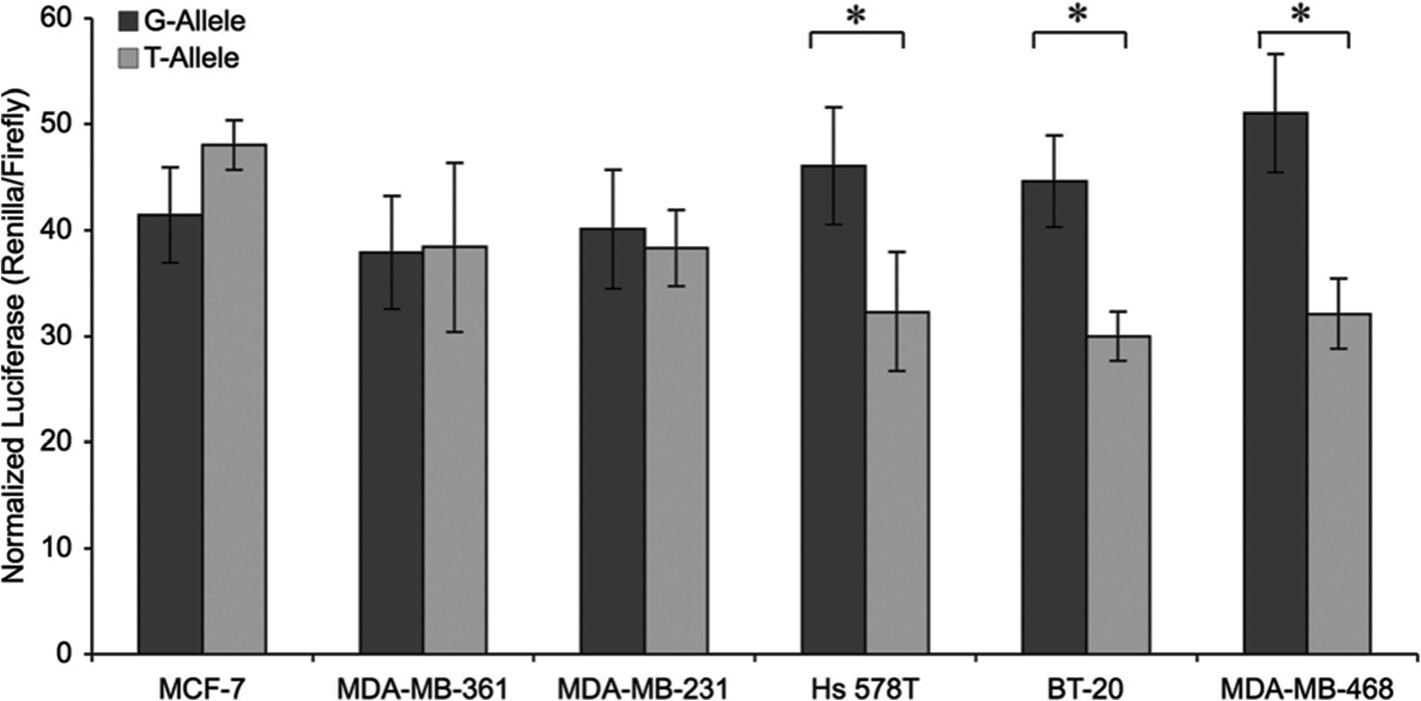
**The impact of the *BRCA1-*3’UTR-variant on luciferase expression in breast cancer cell lines.** Dual luciferase reporters harboring either the non-variant (G-allele, dark grey) or variant (T-allele, light grey) *BRCA1* 3’UTR were transiently transfected into various breast cancer cell lines (as indicated). Following a 16-hour incubation the cells were lysed and luciferase activities were analyzed. Renilla luciferase was normalized to firefly luciferase. T-allele expression was calculated relative to that of the G-allele. Plotted is the mean and standard deviation of 4 independent experiments. *p < 0.05; error bars represent the mean ± standard deviation.

To evaluate if the *BRCA1-*3’UTR-variant alters *BRCA1* gene expression, we generated and tested luciferase reporters containing either the mutant (T) or wild-type (G) *BRCA1* 3’UTR. Reporters were transfected into various breast cancer cell lines and assayed for differences in luciferase gene expression as a surrogate for *BRCA1* expression in the presence or absence of the *BRCA1-*3’UTR-variant. We found that the reporter with the T-allele had decreased luciferase expression by approximately 1.4, 1.5 and 1.8-fold in BT-20, Hs 578 T and MDA-MB-468 triple negative breast cancer cell lines, respectively (Figure 1). We found no significant difference in luciferase expression between the wild-type (G) and mutant (T) alleles in the MDA-MB-361, the MDA-MB-231 or MCF-7 breast cancer cells.

### The BRCA1-3’UTR-variant is associated with decreased BRCA1 gene expression in triple negative breast cancer patients

**Figure 2:**
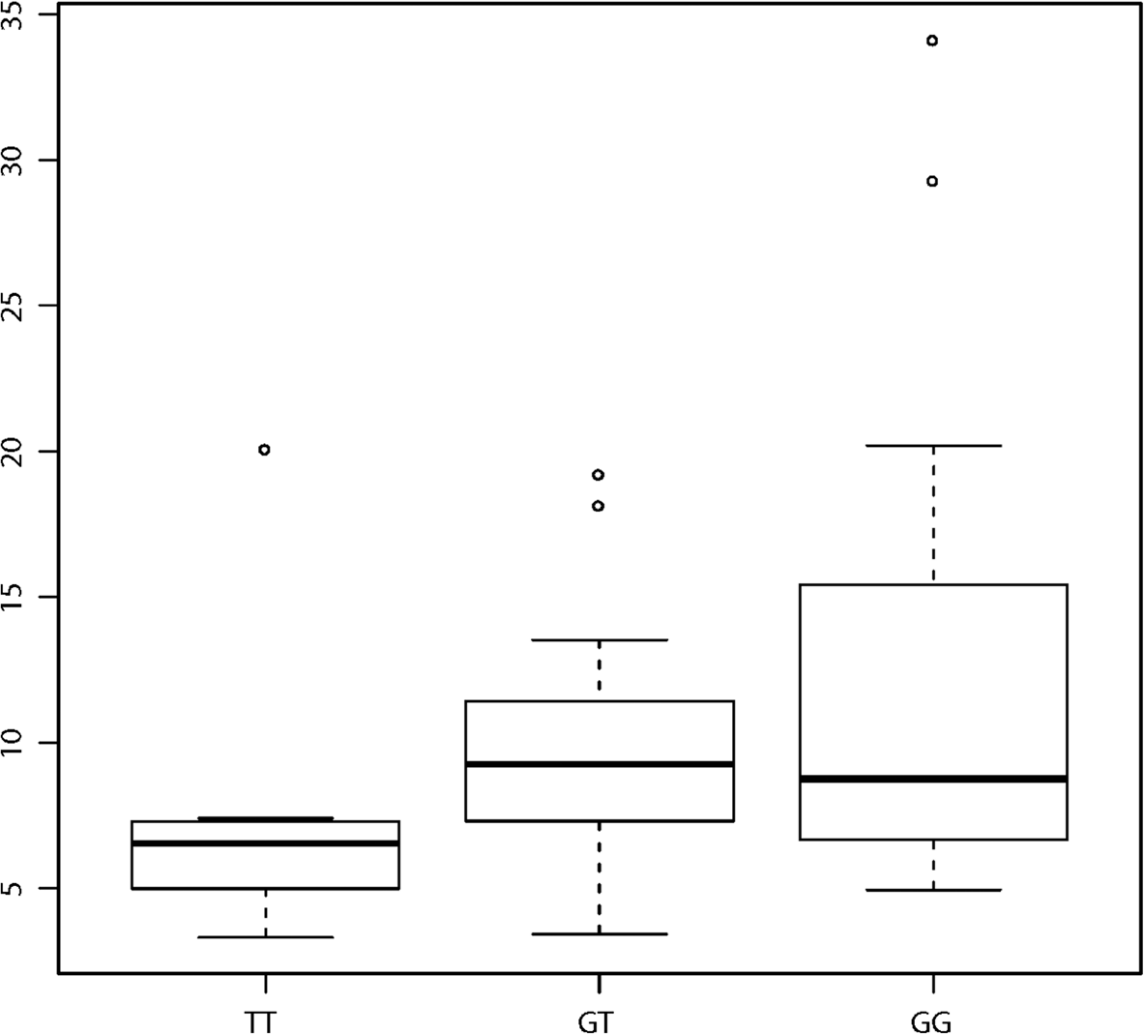
**The *BRCA1-*3’UTR-variant and *BRCA1 *protein staining in CT TNBC patient tumor cores.** Comparison of the degree of *BRCA1* protein staining in a TMA according to respective alleles. A lower level of *BRCA1* staining was noted in the homozygous mutant specimens (TT).

To confirm our *in vitro* findings, we evaluated *BRCA1* protein expression using our CT cohort of triple negative breast cancer patient tumor samples, where *BRCA1* protein staining and the *BRCA1-*3’UTR-variant genotype analysis was available. While protein coding sequence *BRCA1* and *BRCA2* variant status was unavailable for these patients, based on previous work, the *BRCA1* 3’UTR variant is rarely found in patients with coding sequence variants [17]. Even without excluding protein coding sequence mutants, we found *BRCA1* expression was significantly lower in TNBC tumor cores from patients harboring the *BRCA1-*3’UTR-variant (TT) alleles compared to patients harboring hetero and homozygous wild-type (TG and GG) alleles (Figure 2). These findings support the hypothesis that the *BRCA1-*3’UTR-variant is associated with lower *BRCA1* protein expression in TNBC tumors, as was seen *in vitro*.

### Estrogen withdrawal leads to altered gene expression from the BRCA1-3’UTR-variant mutant allele

Based on our findings suggesting that at baseline the *BRCA1-*3’UTR-variant led to differential BRCA expression, we next tested the hypothesis that the *BRCA1-*3’UTR-variant T-allele could be differentially regulated by external cellular events. We chose to study the impact of the presence or absence of estrogen, based on its association with altered expression in TNBC cell lines and tumors. We therefore measured the impact of estrogen withdrawal on our mutant and wild-type luciferase reporters. MCF-7 cells cultured in fulvestrant (an anti-estrogen) or estrogen for 48-hours were transfected with luciferase reporters harboring either the wild-type (G) or mutant (T) *BRCA1* 3’UTR.

**Figure 3:**
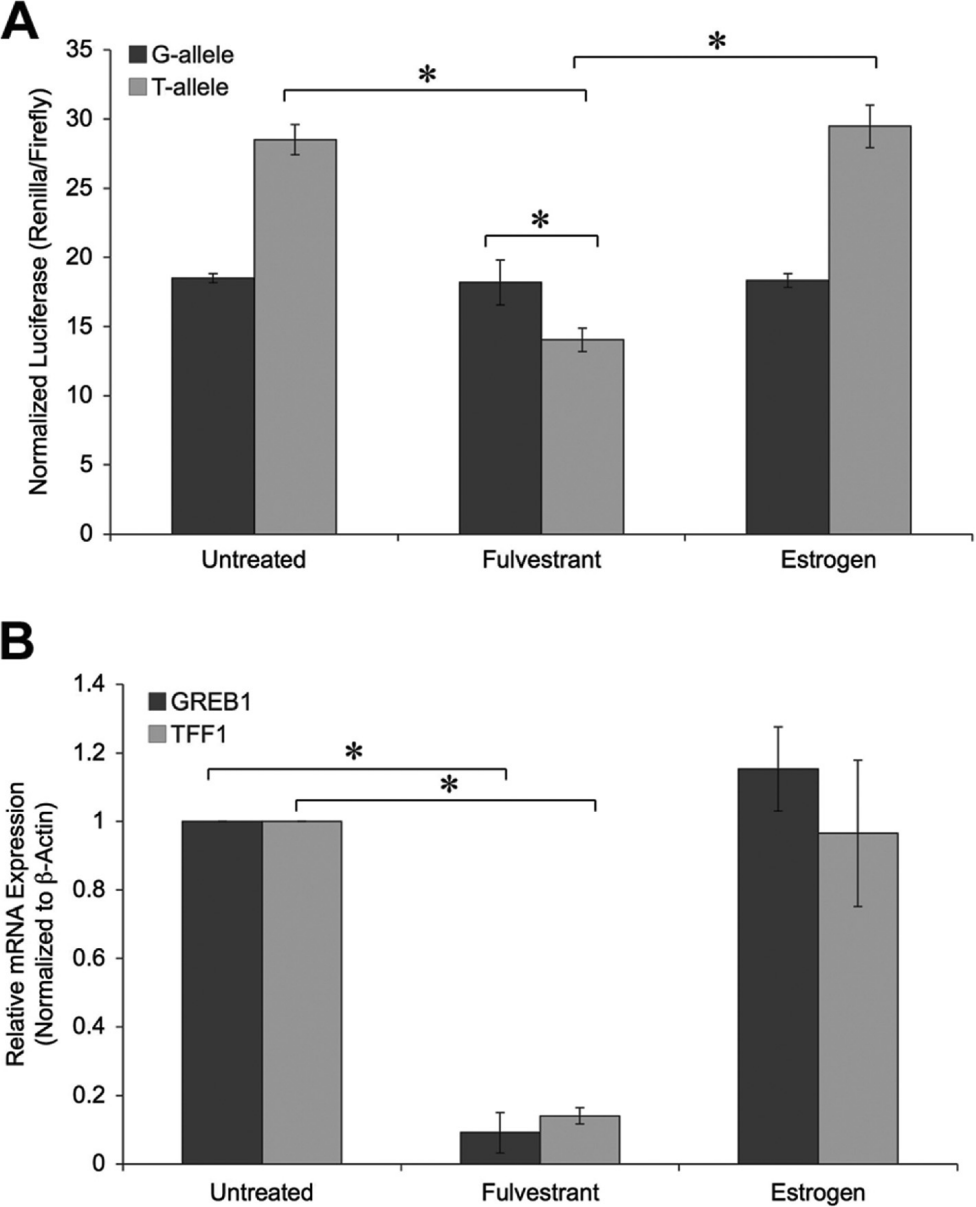
**Expression of the *BRCA1-*3’UTR-variant mutant allele with estrogen withdrawal. A**. MCF-7 cells treated with either 100 nM fulvestrant or estrogen for 48-hours, were transfected with dual luciferase reporter plasmids harboring either the non-variant (G-allele, dark grey) or variant (T-allele, light grey) *BRCA1* 3’UTR. After a 16-hour incubation dual luciferase activities were measured. Renilla luciferase was normalized to firefly luciferase. T-allele expression was calculated to that of the G-allele. Plotted is the mean and standard deviation of at 3 independent experiments. *p < 0.05; error bars represent the mean ± standard deviation. **B**. Total RNA was isolated from cell lysates **(A)** by Trizol extraction. RT-qPCR was utilized to access the effects of fulvestrant and estrogen treatment on mRNA expression of estrogen responsive markers (GREB1 and TFF1). The results were normalized to β-Actin mRNA expression. *p < 0.05; error bars represent the mean ± standard deviation.

We found that estrogen withdrawal resulted in a significant decrease in the expression of the mutant allele, without any impact on the wild-type G-allele, indicating that estrogen withdrawal differentially impacts the expression of the T-allele, leading to down-regulation of luciferase expression in the absence of estrogen. In contrast, we found that the addition of estrogen had no effect on either the non-mutant (G-allele) or mutant allele (T-allele) (Figure 3A). Estrogen depletion was confirmed by RT-PCR analysis of previously described estrogen responsive genes GREB1 [33] and TFF1 [34], which displayed a 10-fold and 7-fold decrease in mRNA expression (respectively) in cells treated with fulvestrant (Figure 3B).

### The association of the BRCA1-3’UTR-variant with breast cancer risk

To determine if there were clinical and biological impacts of the *BRCA1-*3’UTR-variant, we studied a genetically and environmentally homogeneous population, to best control for “context” effects on variant function. We used our case–control analysis of 726 cases and 387 controls from west-Ireland. Clinico-pathological variables of breast cancer cases evaluated in this study and their association with the variant are in Additional file 1. Overall, there was a significant difference in the distribution of the three genotypes across cases and controls (p = 0.033), with a higher proportion of cases displaying the mutant TT and GT genotypes (60[8%] and 318[44%] of 726 cases respectively) compared to controls (29[7%] and 141[36%] of 387 controls respectively). The dominant model was predictive of breast cancer risk compared to controls for all breast cancer patients (OR 1.4, 95% CI 1.1-1.8).

We next evaluated the association of the *BRCA1-*3’UTR-variant across the various breast cancer subtypes. Our cohort was comprised of 519 women with Luminal A breast cancer, 84 with Luminal B disease, 40 with HER2 positive disease and 83 with triple negative breast cancer. 378 (52%) of the 726 breast cancer cases had the variant, with similar prevalence between the subtypes (p = 0.392): Luminal A (279 [54%] of 519 cases), Luminal B (37 [44%] of 84 cases), HER2 (21 [53%] of 40 cases) and triple negative breast cancer (41 [49%] of 83 cases). Comparing the prevalence of the *BRCA1-*3’UTR-variant within respective subtypes with controls, Luminal A breast cancer was most strongly associated with the variant by the dominant model (OR = 1.5, 95% CI 1.1-1.9). This association was not seen with the other subtypes (Additional file 2), but this was likely due to sample size.

Previous work indicated that the homozygous (TT) mutant genotype was associated with triple negative breast cancer in African American patients [17]. Therefore, we evaluated the association of patient/tumor features (age, menopausal status, stage, ER/PR status, and tumor grade) with the homozygous TT variant compared to hetero TG or homozygous GG alleles in all Irish cases. In agreement with this prior study, Irish Caucasian patients with ER/PR negative disease were 2.2 times more likely to carry the homozygous (TT) rs8176318 variant, which was of borderline significance (95% CI 0.98-4.87, p = 0.056).

### The association of the BRCA1-3’UTR-variant with tumor biology and clinical presentation

We next tested the hypothesis that the *BRCA1-*3’UTR-variant may predict altered breast cancer biology in our Irish cohort of patients. We found that both disease stage (p = 0.015) and presence of distant metastasis at presentation (p = 0.037) were significant predictors of the *BRCA1-*3’UTR-variant. Regression analyses of all breast cancer cases evaluating the contributory role of age, menopausal status, tumor grade, stage and ER/PR status in predicting the *BRCA1-*3’UTR-variant was significant only for stage (Table 1). Moreover, patients with metastatic disease (n = 23) at presentation had a four-fold risk of carrying the *BRCA1-*3’UTR-variant compared to Stage I breast cancer patients (p = 0.018, OR 3.73, 95% CI 1.26-11.07). Put differently, 17 (73%) of the 23 patients with metastatic disease at presentation were positive for the *BRCA1-*3’UTR-variant, compared to 349 (51%) of 680 patients without metastatic lesions (p = 0.040, OR 2.7, 95% CI 1.1-6.9) (Table 2). Controlling for other disease variables in a multi-variant model, patients with Stage IV disease were three-fold more likely to have the *BRCA1-*3’UTR-variant compared to all other stages of breast cancer (p = 0.055, OR 2.76, 95% CI 1.0-7.8).

**Table 1:** **Multivariate analysis evaluating the role of patient and pathological factors on the *BRCA1-*** **3’UTR-variant positivity**

**Multivariate analysis**		**p-value**	**OR**	**95% CI**
Age		0.915	1	0.98-1.02
Menopausal status:	Post	0.492	1.29	0.62-2.69
Grade	2	0.493	0.83	0.49-1.41
	3	0.145	0.66	0.38-1.16
Stage	2	0.065	1.48	0.98-2.24
	3	0.169	1.41	0.87-2.29
	4	**0.018**	**3.73**	**1.26-11.07**
ER and/or PR status:	Positive	0.603	0.88	0.56-1.41

**Table 2:** Genotype distribution across metastasis status

**All cases**	**M1**	**M0**	**p-value**	**OR (95% CI)**
TT and GT	17	349	0.040	2.69 (1.05-6.90)
GG	6	331		
**Luminal A cases**				
TT and GT	11	261	0.029	9.86 (1.26-77.01)
GG	1	234		

We further performed regression analysis of Luminal A cases alone, evaluating the effect of patient age, menopausal status, disease stage, tumor stage and grade on the *BRCA1-*3’UTR-variant status. Again we found that the *BRCA1-*3’UTR-variant was significant for disease stage. Patients presenting with Stage IV disease with Luminal A breast cancer had a 10-fold increased risk of carrying the variant compared to patients with Stage I disease (p = 0.033, OR 10.05, 95% CI 1.21-83.52). Presence of distant metastasis at presentation was independently associated with variant positivity, as 11 (92%) of 12 Luminal A patients with metastasis had the variant compared to 261 (53%) of 495 patients without metastasis (p = 0.029, OR 9.9, 95% CI 1.3-77.0) (Table 2). Controlling for other confounding pathological factors in a multivariant model, Stage IV disease in Luminal A cases was again associated with the *BRCA1-*3’UTR-variant compared to all other stages (p = 0.053, OR 7.78, 95% CI 1.0-62.3). In contrast, we found no difference between disease free survival, progression free or overall survival using either a recessive or dominant model (data not shown).

### The BRCA1-3’UTR-variant is not associated with features predicting difficulty in detection

As mammographic screening is initiated at the age of 50 in Ireland, and all women standardly participate, we tested the hypothesis that this mutation might predict difficulty successfully detecting breast cancer using standard screening in these patients, explaining the association with Stage IV presentation. We therefore studied a cohort of women at high risk for breast cancer, with detailed information collected prospectively on health, screening and outcomes from Vermont. Out of this cohort of 369 tested women, 199 had the *BRCA1* 3’UTR variant. As dense breast tissue predicts increased difficulty in tumor detection [35-37], we examined features of mammographic density in this population. We found that women with the *BRCA1* 3’UTR variant were actually significantly less likely to have dense breast tissue compared to non-*BRCA1* 3’UTR mutant patients, when they had an obese BMI (p = 0.0398) (Table 3). To better understand this, we analyzed the relationship between each density category and the *BRCA1* 3’UTR variant. We found that women with the *BRCA1* 3’UTR variant were less likely to have mammograms with scattered fibroglandular density (p = 0.1397) (Table 4 and Additional file 3), which contributes to density. These findings suggest that mammographic screening for women with this variant should be at least as successful in detecting disease as in women without this variant, and thus failed screening is not the explanation for presentation with Stage IV disease.

**Table 3:** Genotype distribution across mammographic density Vermont cohort

**Mammographic density <50%**	**rs8176318 positive**	**rs8176318 negative**	p-value*
Full cohort	93	70	0.2948
Pre-menopausal	49	39	0.7842
Post-menopausal	35	28	0.2530
Normal BMI	26	22	0.7342
Overweight BMI	29	20	0.3115
**Obese BMI**	**36**	**26**	**0.0398**
Menarche age 7-11	16	17	0.7848
Menarche age 12-13	48	33	0.4672
Menarche age ≥14	18	9	0.2123

*P-values represent significance of the association between mammographic density and the presence or absence of rs8176318.

**Table 4:** Genotype distribution across fibroglandular status

**Mammographic density categories**	**rs8176318 positive**	**rs8176318 negative**	p-value*
Extremely dense	17	18	0.5936
Heterogeneously dense	89	82	0.5306
Scattered fibroglandular	66	44	0.1397
Fatty replaced	27	26	0.6576

## Discussion

Here we show for the first time that the rs8176318 G > T 3’UTR variant (the *BRCA1-*3’UTR-variant) is associated with decreased *BRCA1* expression both *in vitro* and *in vivo,* and is impacted by cellular exposure to estrogen. More importantly, we show that this variant predicts aggressive breast cancer biology and stage IV disease, as well as modest increased breast cancer risk in a homogeneous well-characterized west-Irish population. In addition, studying a collection of women at high risk for breast cancer, we found that this variant is associated with features usually considered to improve the ability of mammograms to detect disease (lower mammographic density). These findings suggest that presentation with stage IV disease of *BRCA1-*3’UTR-variant patients is unlikely to be due to the inability to detect disease early, but instead suggests that this variant predicts biologically aggressive disease. These are hypothesis deserving further investigation.

While the findings of increased cancer risk are in agreement with prior reports [16,17], this is the first study evaluating biologic function and clinical associations of the *BRCA1-*3’UTR-variant with the patients that are carriers and develop cancer. While the search for germ-line variants in *BRCA1* have predominantly focused on open-reading-frame variants, increasing evidence is showing that alterations in non-coding regions of genes (such as this variant) explain a proportion of cancer susceptibility, and more importantly play a role in tumor biology and can act as prognostic biomarkers. While the exact biological mechanism leading to altered *BRCA1* expression in *BRCA1-*3’UTR-variant associated tumors is unknown, it is predicted to be a miRNA binding site of miR-20a-3p and miR-5001-3p by target prediction programs including MirSNP and PolymiRTS, and was shown previously to be impacted by miRNA targeting [16]. We hypothesize that this may be more complex, with this region potentially being a landing dock for other RNA binding proteins, and is work that is ongoing but outside of the scope of this proposal.

Diminished expression of *BRCA1* has previously been shown to increase the growth rate of benign and malignant breast tissue [38,39]. In another study, loss of nuclear *BRCA1* expression (using IHC) was significantly associated with high histological grade (p < 0.025) (p < 0.05) [40]. Both of these findings could help explain the association of the *BRCA1-*3’UTR-variant with tumor progression and aggressive phenotype. Interestingly, low *BRCA1* mRNA expression identified in sporadic breast cancer specimens has been associated with development of distant metastasis (p = 0.019) and a shorter disease free interval (p = 0.015) [41]. Additionally, Japanese women whose tumors stained negative for *BRCA1* expression had worse disease free survival than similar patients whose tumors were positive for *BRCA1* staining [42]. Overall, these findings are in agreement with our findings regarding the *BRCA1-*3’UTR-variant, that reduced *BRCA1* expression in the absence of germ-line protein coding sequence variants may be associated with aggressive tumor biology.

Although the *BRCA1-*3’UTR-variant has now been shown to predict a significant increased risk of breast cancer risk in three independent well-characterized cohorts, it is notable that this variant has not been reported from GWAS analyses. We hypothesize that this may be partly due to the association of the *BRCA1-*3’UTR-variant with advanced disease presentation, as patients with Stage IV cancer are generally underrepresented in cohorts that are not comprehensive sequential patient collections, such as the one used in this study, as well as in the Pelletier triple negative cohort study [17]. Another possibility is that because this variant, similar to other identified 3’UTR variants, is altered by “context”, in this case estrogen, which will be altered by body habitus as well as the societal acceptance of hormone replacement therapy, it would make it more likely to be missed in mixed populations such as those used in GWAS studies. For this new class of mutation, 3’UTR variants, the homogeneity and appropriate characterization of the study sample is likely to be much more important than simple sample size.

Our findings suggest a hypothesis where in women with the *BRCA1-*3’UTR-variant, if progressing to an estrogen independent phenotype, their *BRCA1* becomes even less functional, possibly allowing more DNA damage, and perhaps selection for a more aggressive breast cancer genotype. These findings could also indicate that the *BRCA1-*3’UTR-variant becomes the greatest risk for cancer development at the time of estrogen withdrawal, or menopause. While the steps required to lead to breast tumorigenesis in these patients will require studies with *in vitro* and *in vivo* models, this work represents a significant step forward in generating hypotheses about this variant, as well as understanding the role of this variant, and other such variants, in cancer biology.

## Conclusion

Here we show for the first time that the *BRCA1-*3’UTR-variant predicts Stage IV disease, likely due to aggressive tumor biology. The discovery of a meaningful clinical association of the *BRCA1-*3’UTR-variant in breast cancer further highlights the importance of studying such variants in appropriate cohorts to better understand their clinical potential.

## Abbreviations

*BRCA1*: Breast cancer 1, early onset gene; 3’UTR: 3’untranslated region; mRNA: messenger RNA; miRNA: microRNA; Her2: Human epidermal growth factor receptor 2; OR: Odds ratio; CI: Confidence interval; PCR: Polymerase chain reaction; qPCR: quantitative polymerase chain reaction; FISH: Fluorescence in situ hybridization; DFS: Disease free survival; PFS: Progression free survival; OS: Overall survival; TNBC: Triple negative breast cancer; FFPE: Fresh frozen paraffin embedded; TMA: Tissue microarray; ER: Estrogen receptor; PR: Progesterone receptor; RT-qPCR: Reverse transcription quantitative polymerase chain reaction; GREB1: Growth regulated by estrogen in breast cancer 1; TFF1: Trefoil factor 1; GWAS: Genome wide association study.

## Competing interest

JBW is the co-founder of a company that has licensed IP regarding the rs8176318 polymorphism from Yale University.

## Authors’ contributions

JD carried out the genotyping and participated in writing the manuscript. DWS participated in the study design, carried out the luciferase reporter assays and participated in writing the manuscript. CP carried out luciferase reporter assays. RL preformed the AQUA analysis. CS, CC, KLG, TM, LH participated in patient sample and database curation. DW and JN carried out the statistical analysis. MK, NM participated in developing the study design. TR and MW analyzed the Vermont samples and weighed in on the interpretation. JBW participated in developing the study design, coordination of collaborations and patient sample acquisition and helped write the manuscript. All authors read and approved the final manuscript.

## Pre-publication history

The pre-publication history for this paper can be accessed here:

http://www.biomedcentral.com/1471-2407/14/421/prepub

## Supplementary Material

Additional file 1 Clinicopathological characteristics of breast cancer cases. Click here for file

Additional file 2 Association between subtypes and controls. Click here for file

Additional file 3 Mammographic density categories. Click here for file
