# Supplementary material for: A germline mutation in the BRCA1 3’UTR predicts Stage IV breast cancer
Source: BMC Cancer. 2014 Jun 10;14:421. doi: 10.1186/1471-2407-14-421 (PMC4059881; doi:10.1186/1471-2407-14-421)
Supplement: Additional file 1 — Clinicopathological characteristics of breast cancer cases. [file 1471-2407-14-421-S1.doc]

**Additional File 1**: Clinicopathological characteristics of breast cancer cases

|  | |  | | **Overall** | **rs8176318 (TT+TG)** | **rs8176318**  **(GG)** | **p-value** |
| --- | --- | --- | --- | --- | --- | --- | --- |
| Study population (N,%) | | | | 726 (100) | 378 (52) | 348 (48) |  |
| Age (mean ±SD) | | | | 54 ±11 | 54 ±12 | 54 ±11 | 0.407 |
| Menopausal Status (N,%) | | Pre  Post | | 286 (44)  365 (56) | 145 (43)  195 (57) | 141 (45)  170 (55) | 0.762 |
| Tumor size (mm ±SD) | | | | 28.9 ±18.3 | 29.5 ±18.5 | 28.3 ±18.2 | 0.404 |
| Tumor grade (N, %) | | 1  2  3 | | 81 (13)  308 (50)  222 (37) | 46 (15)  161 (51)  109 (34) | 35 (12)  147 (50)  113 (38) | 0.476 |
| pN (N,%) | | 0  1  2  3 | | 327 (49)  203 (30)  95 (14)  43 (7) | 157 (46)  113 (33)  50 (14)  25 (7) | 170 (53)  90 (28)  45 (14)  18 (5) | 0.283 |
| M (N,%) | | 0  1 | | 643 (97)  23 (3) | 325 (95)  17 (5) | 318 (98)  6 (2) | **0.037** |
| Stage (N,%) | | 1  2  3  4 | | 197 (28)  325 (46)  158 (23)  23 (3) | 87 (24)  175 (48)  87 (24)  17 (4) | 110 (33)  150 (44)  71 (21)  6 (2) | **0.015** |
| ER and/or PR status (N,%) | Negative  Positive | | | 123 (17)  603 (83) | 62 (16)  316 (84) | 61 (18)  287 (82) | 0.686 |
| Subtype (N,%) | Luminal A  Luminal B  HER2  TNBC | | | 519 (71)  84 (12)  40 (6)  83 (11) | 279 (74)  37 (10)  21 (5)  41 (11) | 240 (69)  47 (14)  19 (5)  42 (12) | 0.392 |
| Disease Recurrence (N,%) | | | Yes  No | 125 (18)  566 (82) | 70 (20)  286 (80) | 55 (16)  280 (84) |  |
| Disease Free Survival  (months ±SD) | | | | 48 ±48 | 47 ±43 | 50 ±53 | 0.227 |
| Site of primary metastasis | Bone  Other | | | 16 (73)  6 (27) | 11 (67)  5 (33) | 5 (83)  1 (17) | 0.479 |
| Disease Progression | Yes  No | | | 9 (50)  9 (50) | 8 (57)  6 (43) | 2 (50)  2 (50) |  |
| Progression Free Survival (months ±SD) | | | | 28 ±22 | 27 ±21 | 32 ±27 | 0.361 |
| Death | Yes  No | | | 20 (3)  670 (97) | 12 (3)  343 (97) | 8 (2)  327 (98) |  |
| Overall Survival (months ±SD) | | | | 52 ±54 | 50 ±50 | 53 ±58 | 0.305 |
