# Supplementary material for: A germline mutation in the BRCA1 3’UTR predicts Stage IV breast cancer
Source: BMC Cancer. 2014 Jun 10;14:421. doi: 10.1186/1471-2407-14-421 (PMC4059881; doi:10.1186/1471-2407-14-421)
Supplement: Additional file 2 — Association between subtypes and controls. [file 1471-2407-14-421-S2.doc]

**Additional File 2**: Association between subtypes and controls

| **rs8176318**  **Genotype** | **Luminal A vs Controls** | | **Luminal B vs Controls** | | **HER2 vs Controls** | | **TNBC vs Controls** | |
| --- | --- | --- | --- | --- | --- | --- | --- | --- |
|  | OR  (95%CI) | p-  value | OR (95%CI) | p-value | OR (95%CI) | p-value | OR (95%CI) | p-value |
| TT vs GG | 1.3  (0.8-2.1) | 0.41 | 1.0  (0.4-2.4) | 0.9234 | 2.4  (0.9-6.4) | 0.1551 | 1.2  (0.5-3.0) | 0.8036 |
| GT vs GG | **1.5**  **(1.2-2.0)** | **0.0035** | 1.0  (0.6-1.7) | 0.9532 | 1.2  (0.6-2.5) | 0.7223 | 1.2  (0.8-2.1) | 0.4623 |
| TT/GT vs GG | **1.5**  **(1.1-1.9)** | **0.0042** | 1.0  (0.6-1.6) | 0.9840 | 1.4  (0.7-2.7) | 0.3837 | 1.2  (0.8-2.0) | 0.4310 |
