# Supplementary material for: A germline mutation in the BRCA1 3’UTR predicts Stage IV breast cancer
Source: BMC Cancer. 2014 Jun 10;14:421. doi: 10.1186/1471-2407-14-421 (PMC4059881; doi:10.1186/1471-2407-14-421)
Supplement: Additional file 3 — Mammographic density categories. [file 1471-2407-14-421-S3.doc]

**Additional File 3:** Mammographic Density Categories

|  | **rs8176318 positive** | **rs8176318 negative** |
| --- | --- | --- |
| Extremely Dense | 17 | 18 |
| Not Extremely Dense | 182 | 152 |

p=0.5936

|  | **rs8176318 positive** | **rs8176318 negative** |
| --- | --- | --- |
| Heterogeneously Dense | 89 | 82 |
| Not Heterogeneously Dense | 110 | 88 |

p=0.5306

|  | **rs8176318 positive** | **rs8176318 negative** |
| --- | --- | --- |
| Scattered Fibroglandular | 66 | 44 |
| Not Scattered Fibroglandular | 133 | 125 |

p=0.1397

|  | **rs8176318 positive** | **rs8176318 negative** |
| --- | --- | --- |
| Fatty Replaced | 27 | 26 |
| Not Fatty Replaced | 172 | 144 |

**p=0.6576**

|  | **rs8176318 positive** | **rs8176318 negative** |
| --- | --- | --- |
| Upper 50% Density | 106 | 100 |
| Lower 50% Density | 93 | 70 |

p=0.2948

| *Premenopausal Women* | **rs8176318 positive** | **rs8176318 negative** |
| --- | --- | --- |
| Upper 50% Density | 79 | 58 |
| Lower 50% Density | 49 | 39 |

p=0.7842

| *Postmenopausal Women* | **rs8176318 positive** | **rs8176318 negative** |
| --- | --- | --- |
| Upper 50% Density | 21 | 28 |
| Lower 50% Density | 35 | 28 |

p=0.2530

| *Normal BMI* | **rs8176318 positive** | **rs8176318 negative** |
| --- | --- | --- |
| Upper 50% Density | 73 | 54 |
| Lower 50% Density | 26 | 22 |

p=0.7342

| *Overweight BMI* | **rs8176318 positive** | **rs8176318 negative** |
| --- | --- | --- |
| Upper 50% Density | 23 | 26 |
| Lower 50% Density | 29 | 20 |

p=0.3115

| *Obese BMI* | **rs8176318 positive** | **rs8176318 negative** |
| --- | --- | --- |
| Upper 50% Density | 6 | 14 |
| Lower 50% Density | 36 | 26 |

p=0.0398

| *Menarche 7-11* | **rs8176318 positive** | **rs8176318 negative** |
| --- | --- | --- |
| Upper 50% Density | 12 | 10 |
| Lower 50% Density | 16 | 17 |

p=0.7848

| *Menarche 12-13* | **rs8176318 positive** | **rs8176318 negative** |
| --- | --- | --- |
| Upper 50% Density | 62 | 55 |
| Lower 50% Density | 48 | 33 |

p=0.4672

| *Menarche ≥14* | **rs8176318 positive** | **rs8176318 negative** |
| --- | --- | --- |
| Upper 50% Density | 20 | 21 |
| Lower 50% Density | 18 | 9 |

p=0.2123

| *Premenopausal* | **rs8176318 positive** | **rs8176318 negative** |
| --- | --- | --- |
| Top 25% Density | 16 | 12 |
| Bottom 25% Density | 15 | 13 |

p=1.000

| *Postmenopausal* | **rs8176318 positive** | **rs8176318 negative** |
| --- | --- | --- |
| Top 25% Density | 1 | 3 |
| Bottom 25% Density | 11 | 12 |

p=0.6051

|  | **rs8176318 positive** | **rs8176318 negative** |
| --- | --- | --- |
| Top 25% Density | 17 | 18 |
| Bottom 25% Density | 27 | 26 |

p=1.000
